# Hepatitis E, a Vaccine-Preventable Cause of Maternal Deaths

**DOI:** 10.3201/eid1809.120241

**Published:** 2012-09

**Authors:** Alain B. Labrique, Shegufta S. Sikder, Lisa J. Krain, Keith P. West, Parul Christian, Mahbubur Rashid, Kenrad E. Nelson

**Affiliations:** Johns Hopkins Bloomberg School of Public Health, Baltimore, Maryland, USA (A.B. Labrique, S.S. Sikder, L.J. Krain, K.P. West, P. Christian, K.E. Nelson);; and JiVitA Maternal and Child Health and Nutrition Research Project, Gaibandha, Bangladesh (M. Rashid)

**Keywords:** hepatitis E, hepatitis, viruses, humans, pregnancy, pregnancy complications, maternal mortality, vaccines, hepatitis vaccines, immunization, vaccination, population surveillance, Asia, Bangladesh, Nepal, China, developing countries

## Abstract

Hepatitis E virus (HEV) is a major cause of illness and of death in the developing world and disproportionate cause of deaths among pregnant women. Although HEV vaccine trials, including trials conducted in populations in southern Asia, have shown candidate vaccines to be effective and well-tolerated, these vaccines have not yet been produced or made available to susceptible populations. Surveillance data collected during 2001–2007 from >110,000 pregnancies in a population of ≈650,000 women in rural Bangladesh suggest that acute hepatitis, most of it likely hepatitis E, is responsible for ≈9.8% of pregnancy-associated deaths. If these numbers are representative of southern Asia, as many as 10,500 maternal deaths each year in this region alone may be attributable to hepatitis E and could be prevented by using existing vaccines.

## Abstract 2

These deaths are substantial and could be prevented by commercial vaccine.

We sometimes remark that hepatitis E is so neglected that it fails to make the short list of neglected tropical diseases (*1*). Since the identification of hepatitis E virus (HEV) as a distinct viral agent in 1983 (*2*) and its subsequent cloning and sequencing in 1990–1991 (*3*), much has been learned about the still-perplexing epidemiology of this virus, which is now the leading cause of acute viral hepatitis globally (*4*). The landmark feature of HEV genotype 1, which predominates in populations in the greater Ganges floodplains of southern Asia, is increased deaths in pregnant women (*5*). The poorly understood pregnancy-associated case-fatality rate of hepatitis E, ranging from 7% to 40%, was noted in the earliest confirmed hepatitis E epidemic in 1955 (*6*) and has since been repeatedly confirmed by studies of sporadic cases and outbreaks.

Rein et al. (*7*) recently published their modeled estimates of the global incidence of HEV infections and associated deaths in 2005, suggesting >20 million incident infections and 3.3 million cases of hepatitis E per year, and a 20% probability of death in infected pregnant women. Some of these models were based, in part, on rigorous epidemiologic data collected by our group in a large research population, the Matlab demographic surveillance site in rural Bangladesh (*8**,**9*). Establishing reliable estimates of national and regional disease burden has been challenging because of the lack of standard assays for hepatitis E testing and substantial variability in the quality of commercial assays (*10**,**11*). However, it is clear that populations who show the highest incidence of HEV-associated illnesses and deaths are not benefiting from existing HEV vaccines or public health interventions.

In the mid-1990s, success with HEV vaccination in primates led to phase I trials of a recombinant HEV vaccine in 88 adults at the Walter Reed Army Institute of Research (*12*). During 2001–2004, a phase II clinical trial supported by the US Army and GlaxoSmithKline (Brentford, UK) was conducted in 1,794 predominantly male military conscripts from Nepal (*13*), but this trial was embroiled in controversy from its inception (*14**,**15*). An earlier community trial had been forcibly terminated because political opposition claimed the vaccine would be unaffordable to populations in which the vaccine was to be tested (*16*). Ultimately, this vaccine showed an efficacy of 88.5% after the first dose, which increased to >95.5% after 3 doses (*13*). These data were not published until 2007, three years after the end of the trial.

Unfortunately, GlaxoSmithKline did not pursue the manufacture of this vaccine, despite a growing body of evidence that confirms the substantial incidence of HEV infections in resource-limited sub-Saharan Africa, Asia, and South America and recent data that indicate an unrecognized silent epidemic of autochthonous HEV infections and illnesses across most of eastern Asia and central and western Europe (*17**–**19*). Recent evidence from our group suggests that HEV may be circulating widely in the United States, although the attributable disease incidence seems low (*20*).

In 2005, researchers at Xiamen University in Fujian, China, began working on a new recombinant HEV vaccine to address growing concerns about the increasing regional incidence of HEV infections. HEV 239 was tested in a randomized, controlled trial involving 112,604 participants in Jiangsu Province, where genotypes 3 and 4 are predominant. The vaccine showed an efficacy >99% in preventing clinical hepatitis E among persons who completed the full 3-dose series of HEV 239 (n = 48,693) compared with placebo (a commercial hepatitis B vaccine, n = 48,663) (*21*). On January 23, 2012, we learned that this vaccine has been licensed for production and sale by the State Food and Drug Administration of China (*22*).

The initial use of this vaccine will probably be in high-risk populations within China. However, the manufacturer (Xiamen Innovax Biotech Co. Ltd., Xiamen, China) has expressed its intent to make the vaccine available in other regions (*23**,**24*). Demonstration of the effectiveness and safety of this vaccine in diverse settings is urgently needed. The vaccine has yet to be tested in high-need populations in which genotype 1 predominates. However, the fact that the capsid protein used in the vaccine was derived from the genotype 1 Burma reference strain (*25*) gives reason for optimism that it will be effective in these populations.

Data on the effectiveness of this vaccine in pregnant women are scant because of the exclusion of pregnant women in the trial in China. Post hoc analysis of 68 trial participants whose pregnancies were detected after receiving 1–3 doses of vaccine or placebo did not raise any obvious concerns (*26*). However, targeted safety, immunogenicity, and efficacy trials in pregnant women are warranted. Given the years of commercial inactivity after the determination of efficacy of previous HEV vaccines, the fact that an HEV vaccine may soon be commercially available in >1 HEV-endemic country is a major milestone on the road toward protecting vulnerable women in disease-endemic areas from HEV infection, fetal loss, and neonatal death or even maternal deaths.

To further underscore the urgency of action in this domain, we now highlight some findings from our ongoing work within the JiVitA Maternal and Child Health and Nutrition Research Site in Gaibandha, Bangladesh. Since 2001, we have been conducting prospective pregnancy surveillance in a northwestern rural population, as part of ongoing randomized community trials that aim to reduce maternal and neonatal deaths (*27**–**29*). In 3 consecutive trials, we have enrolled >110,000 pregnant women from a catchment population of ≈650,000. The details of this field site and these research activities have been reported (*28**,**29*). Study activities were reviewed and approved by the Johns Hopkins Bloomberg School of Public Health Committee on Human Research and by the Bangladesh Medical Research Council.

In this context, 1,091 deaths in women of reproductive age (245 during pregnancy or within 42 days postpartum) were recorded during August 2001–August 2007 by our surveillance system. Verbal autopsies by trained physicians identified hepatitis, hepatic failure, or jaundice as the primary cause of death in 19 (7.8%) of 245 pregnancy-related deaths and 61 (7.2%) of 846 nonpregnant deaths recorded. Overall, hepatitis-like illness was suggested as a direct or underlying cause in 24 (9.8%) of 245 pregnancy-related and 81 (9.6%) of 846 non–pregnancy-related deaths (Table). In another recent study, Khatun et al. (*30*) conducted verbal autopsies for 260 neonatal and 93 maternal deaths in populations in slums of urban Dhaka, Bangladesh. In that setting, viral hepatitis was reported to be the underlying cause for 11% of maternal deaths. In addition, an epidemic of HEV was documented in the slums at the time of the study (*30*).

**Table:** Contribution of hepatitis and hepatitis-like symptoms as direct or underlying causes of death in women of reproductive age, northwestern Bangladesh, 2001–2007

Cause of death	Pregnancy-related deaths,* no. (%)	Non–pregnancy-related deaths, no. (%)	All deaths in enrolled women of reproductive age, no. (%)
All	245 (100.0)	846 (100.0)	1,091 (100.0)
Direct			
Hepatitis	19 (7.8)	61 (7.2)	80 (7.3)
Underlying			
Hepatitis	3 (1.2)	10 (1.2)	13 (1.2)
Jaundice	2 (0.8)	10 (1.2)	12(1.1)
Combined	24 (9.8)	81 (9.6)	105 (9.6)

*Defined as deaths of women who were pregnant or within 42 d of termination of the pregnancy, irrespective of the cause of death.

Verbal autopsy results and other epidemiologic evidence also implicate hepatitis E as the probable cause of most deaths from sporadic hepatitis-like illness in the rural JiVitA cohort, with sudden onset of symptoms in the days or weeks before death. Although our surveillance system did not enable laboratory-based differential diagnosis of individual cases, hepatitis A, B, and C are unlikely to play a major role in deaths in this population of women. Clinical studies of acute liver failure in Bangladesh have shown HEV to be the main etiologic agent responsible (*31**–**33*).

Serosurveys in rural Bangladesh have demonstrated nearly universal exposure to hepatitis A virus during childhood, with subsequent life-long immunity, implying an extremely low probability of symptomatic hepatitis A virus infection during pregnancy. Hepatitis C virus is rare in low-risk rural populations (prevalence of IgG against hepatitis C virus ≈1.5%) (*8*). Hepatitis B virus is endemic to rural Bangladesh, but the incidence of infection and illness are also low (*9*). Therefore, hepatitis E remains the most likely etiologic agent for these fatal hepatitis-like illnesses in pregnant women.

If these numbers are, as we suspect, representative of women of reproductive age and of the incidence of hepatitis throughout Bangladesh, as many as 1,180 of the ≈12,000 deaths that occur during pregnancy and soon after childbirth each year in Bangladesh (*34*) may be attributable to hepatitis E. If we apply these proportions to southern Asia, <10,500 of the estimated annual 109,000 pregnancy-related deaths in southern Asia (*35*) may be attributable to hepatitis E. Furthermore, the substantial contribution of hepatitis-like illness to deaths in nonpregnant women of reproductive age in this population deserves careful attention.

Hepatitis E should no longer be considered an obscure, newly emerging virus. More than 3 decades of study attest to its global dispersion and mortality rate. Nonetheless, etiologic surveillance across countries must be strengthened. Our population-based surveillance data suggest that ≈10% of deaths observed in pregnancy and in women of reproductive age in nonepidemic conditions could be attributable to HEV. With the availability of 2 tested efficacious vaccines, we must consider judicious and timely implementation of such interventions, where appropriate, to avoid a substantial portion of preventable deaths in these resource-limited settings.
